# The effectiveness and safety of treatments used for polycystic ovarian syndrome management in adolescents: a systematic review and network meta-analysis protocol

**DOI:** 10.1186/s13643-015-0105-4

**Published:** 2015-09-23

**Authors:** Reem A. Al Khalifah, Iván D. Flórez, Brittany Dennis, Binod Neupane, Lehana Thabane, Ereny Bassilious

**Affiliations:** Department of Clinical Epidemiology & Biostatistics, McMaster University, Juravinski Site, G Wing, 2nd Floor, 711 Concession Street, Hamilton, ON L8V 1C3 Canada; Department of Pediatrics, Division of Endocrinology and Metabolism, McMaster University, Hamilton, Ontario Canada; Department of Pediatrics, King Saud University, Riyadh, Saudi Arabia; Department of Pediatrics, University of Antioquia, Medellín, Colombia; Department of Pediatrics, McMaster University, Hamilton, Ontario Canada; Department of Pediatrics and Anesthesia, McMaster University, Hamilton, Canada

**Keywords:** Adolescents, Polycystic ovarian syndrome, Hirsutism, Menstrual irregularity, Acne, Body mass index, Dysglycaemia

## Abstract

**Background:**

Polycystic ovarian syndrome (PCOS) is a common reproductive endocrine disease that is seen among adolescent women. Currently, there is limited evidence to support treatment options leading to considerable variation in practice among healthcare specialists. The objective of this study is to review and synthesize all the available evidence on treatment options for PCOS among adolescent women.

**Methods/design:**

We will conduct a systematic review of all randomized controlled trials evaluating the use of metformin, oral contraceptive pills as monotherapy, or as combination with pioglitazone, spironolactone, flutamide, and lifestyle interventions in the treatment of PCOS in adolescent women ages 11 to 19 years. The primary outcome measures are menstrual regulation and change hirsutism scores. The secondary outcome measures include acne scores, prevalence of dysglycaemia, BMI, lipid profile, total testosterone level, and adverse events. We will perform literature searches through Ovid Medline, Ovid Embase, and Cochrane Central Register of Controlled Trials (CENTRAL), and gray literature resources. Two reviewers will independently screen titles and abstracts of identified citations, review the full texts of potentially eligible trials, extract information from eligible trials, and assess the risk of bias and quality of the evidence independently. Results of this review will be summarized narratively and quantitatively as appropriate. We will perform a multiple treatment comparison using network meta-analysis to estimate the pooled direct and indirect effects for all PCOS interventions on outcomes if adequate data is available.

**Discussion:**

PCOS treatment poses a clinical challenge to the patients and physicians. This is the first systematic review and network meta-analysis for PCOS treatment in adolescents. We expect that our results will help improve patient care, unify the treatment approaches among specialists, and encourage research for other therapeutic options.

**Systematic review registration:**

PROSPERO CRD42015016148

## Background

Polycystic ovarian syndrome (PCOS) is a common reproductive endocrine disease encountered among adolescents and young women [1]. Its prevalence varies between 1.8 and 15 % depending on the diagnostic criteria used and ethnicity [1–3]. Patients with PCOS can present with a constellation of symptoms including chronic anovulation (amenorrhea, oligomenorrhea, irregular menstrual cycles), clinical features of hyperandrogenism (acne and hirsutism), biochemical evidence of hyperandrogenism, polycystic ovaries on ultrasound, and features of metabolic syndrome. Oligomenorrhea is the presenting feature in about 75 % of cases [4], while hirsutism and acne are present in 60–70 % of cases and contribute to psychological distress in adolescent patients [4, 5].

Three different diagnostic criteria have been used for the diagnosis of PCOS: the National Institutes of Health (NIH), the Rotterdam, and the Androgen Excess Society Criteria [6–8]. All of them require the presence of menstrual cycle disturbance and presence of clinical and/or biochemical hyperandrogenism, while the last two require the presence of polycystic ovarian morphology on ultrasound [6, 7]. To date, the preferred diagnostic criteria in adolescents are the NIH criteria [9, 10].

The etiology of PCOS is complex and not well understood. Primary intrinsic ovarian pathology in combination with hypothalamic–pituitary–ovarian axis abnormalities may lead to increased ovarian androgen secretion [11, 12]. Insulin resistance with compensatory hyperinsulinemia may also play a role as it can lead to direct stimulation of ovarian and adrenal androgen secretion, which leads to decreased hepatic sex hormone binding globulin synthesis and therefore, to an increased bioavailability of free testosterone level [11–14]. Insulin resistance is involved in the development of cardiometabolic disturbances such as dysglycaemia, hyperlipidemia, and obesity [15–17], and it has been described that between 18 and 24 % of adolescents with PCOS have some degree of abnormal glucose metabolism [18–20]. These patients are at increased risk of type 2 diabetes, hypertension, myocardial infarction, angina, and psychiatric diseases [21, 22] in addition to gynecological and obstetrical complications, such as infertility, higher rate for pregnancy loss, gestational diabetes, premature delivery, as well as gynecological and non-gynecological cancers [22–26]. In addition to the aforementioned co-morbidities, patients with PCOS experience a low perceived health quality over lack of symptom improvement, primarily with weight control, hirsutism, acne, menstrual irregularity, and infertility as inferred from qualitative studies [27–29].

Optimal first line treatment of PCOS in adolescents remains controversial. Current Endocrine Society treatment guidelines first recommend lifestyle changes (dietary and exercise modification) followed by either oral contraceptive pills (OCP) to control symptoms of hyperandrogenism or metformin therapy in patients with impaired glucose tolerance or features of metabolic syndrome [10]. However, there is significant variability in clinical practice, depending on whether the physician and patient’s primary goal of treatment is to treat the symptoms of hyperandrogenism or the features of metabolic syndrome [30, 31]. Additionally, in clinical practice anti-androgenic medications such as spironolactone, flutamide, and insulin sensitizing agents such as pioglitazone are used as add-on therapy when OCP or metformin fail to produce the clinically desired outcomes [4, 31], yet the Endocrine Society guidelines do not comment on their use in the adolescent population.

To date, there is one systematic review and meta-analyses in adolescents (in press) that identified low number of low quality evidence from head-to head trials and identified large number of trials that compared metformin to placebo, OCP to placebo, and other PCOS combination therapy [32]. A traditional meta-analysis can only evaluate the direct treatment efficacy of two treatment approaches at a time while a network meta-analysis can provide effect estimates for all direct and indirect treatment comparisons [33]. Therefore, we aim to conduct a network meta-analysis to address the following objectives: (1) assess the effectiveness and safety of using metformin and OCP as monotherapy in adolescents with PCOS; (2) assess the effectiveness and safety of using metformin and/or OCP in combination with pioglitazone, spironolactone, flutamide, and lifestyle interventions, as evaluated across multiple outcomes such as menstrual cycle regulation, improvement in clinical and or biochemical evidence of hyperandrogenism, and metabolic profile in adolescents with PCOS; (3) evaluate the effectiveness of different formulations of OCPs on hirsutism and acne scores.

## Methods/design

This systematic review and network meta-analysis protocol is registered on PROSPERO International prospective register of systematic reviews (CRD42015016148). The report will comply with the Preferred Reporting Items for Systematic Review and Network Meta-Analysis Protocols (PRISMA-P) [34].

### Eligibility criteria

The search for studies will be limited to randomized clinical trials (RCT) (including all designs such as crossover, cluster, and patient-randomized clinical trials) assessing the efficacy, effectiveness, or safety of different regimen for the treatment of PCOS that enrolled adolescent girls ages 11–19 years. The definition of adolescent age group is based on the widely accepted World Health Organization definition for adolescent [35]. Studies that include both adolescents and adults participants will be included in the review, and upon contact, we will ask authors to provide separate data for the adolescent participants. If we are unable to obtain this information, we will include the study and we will conduct subgroup analyses in order to assess the difference between studies which included only adolescents and studies which included both adolescents and adults. Sub-studies or secondary analysis of reported eligible studies will be excluded to avoid duplication.

The diagnosis of PCOS will be based on the known PCOS diagnostic criteria: Endocrine Society Guidelines, NIH criteria, Rotterdam criteria, and the Androgen Excess Society criteria [6, 7, 10]. We will exclude studies that included normal control participants or patients with other causes of oligomenorrhea or hyperandrogenism, such as hyperprolactinemia, thyroid dysfunction, androgen secreting tumors, or late-onset congenital adrenal hyperplasia.

We will include studies that evaluated single and/or combined interventions, at any dose, such as metformin, OCP, pioglitazone, spironolactone, flutamide, and lifestyle interventions. In order to be included, the study will have had to report the effectiveness of one of these interventions and the intervention effect on one or more of the outcomes of interest.

Our primary outcomes are menstrual cycle regulation and hirsutism scores. The secondary outcomes include acne scores, prevalence of dysglycaemia, BMI, total testosterone level, lipid profile (triglyceride, total cholesterol, LDL, HDL), and adverse events; Table 1 shows the definitions of outcome measures. We chose not to report on pregnancy outcomes because it necessities changing the scope of the review to involve fertility induction medications. Hence, we will exclude studies that only used fertility induction medications and which primary outcome of interest was pregnancy.

**Table 1:** Outcome measures

Outcome	Measurement of variable (units)	Statistical estimates and measurement of association of this outcome
Menstrual regulation	Number of girls achieved regular menses	Rate ratio
	Number of cycles per year	Mean difference ± SD
Hirsutism	Ferriman Gallawey score	Mean difference ± SD
Acne scores	Lesion counting or grading	Standardized mean difference ± SD
Dysglycaemia	The rate of occurrence of T2DM, impaired glucose tolerance, and impaired fasting glucose assessed by oral glucose tolerance test and/or fasting blood glucose, and/or HBA1c	Rate ratio
BMI	kg/m^2^	Mean difference ± SD
Total testosterone level	ng/ml	Mean difference ± SD
Total cholesterol	mg/dl	Mean difference ± SD
LDL	mg/dl	Mean difference ± SD
HDL	mg/dl	Mean difference ± SD
Triglyceride	mg/dl	Mean difference ± SD
Adverse events	Number of girls developed:	OR, rate ratio
	1- GI: all GI related adverse events:	
	• Nausea	
	• Vomiting	
	• Diarrhea	
	• Constipation	
	• Abdominal pain	
	• Flatulence	
	• Gastritis	
	• GI bleeding	
	2- thrombosis: all vascular events related to thrombus formation such as:	
	• Deep venous thrombosis	
	• Stroke	
	• Myocardial infarction	
	• Pulmonary embolism	
	3- serious: adverse events that are of major morbidity such as:	
	• Any bleeding not including GI bleed	
	• Lactic acidosis	
	• Liver failure	
	• Renal failure	
	• Vasculitis	
	• Electrolyte imbalance	
	• Agranulocytosis	
	• Photosensitivity	
	• Hypertension	
	• Pancreatitis	
	• Anaphylaxis	
	• Chorea	
	• Depression	
	4- minor: adverse events that are of minor morbidity such as:	
	• Headache	
	• Fatigue	
	• Beast tenderness	
	• Vaginal bleeding (spotting)	
	• Edema	
	• Weight gain	
	• Metallic taste	
	• Muscle cramp	
	• Rash	
	• Fever	
	• Hot flashes	
	• Glucose intolerance	
	• Infection	

*OR* odds ratio, *T2DM* type 2 diabetes mellitus, *BMI* body mass index, *LDL* low-density lipoprotein, *HDL* high-density lipoprotein, *GI* gastrointestinal

### Data sources and search strategy

We performed literature search through Ovid MEDLINE, Ovid EMBASE, and Cochrane Central Register of Controlled Trials (CENTRAL) from the database inception to January 2015 using combination of controlled terms, i.e., Medical Subject Heading (MeSH), Emtree terms, and free-text terms with various synonyms for polycystic ovarian syndrome (PCOS), adolescent, metformin, pioglitazone, oral contraceptive pills, flutamide, and lifestyle interventions (Appendix).

We used the randomized controlled trial filter created from McMaster University for Ovid Embase platform and the Cochrane library filter for Ovid Medline platform [36, 37]. These filters provide a good balance between sensitivity and specificity. Our search strategy was developed in liaison with an experienced librarian. No language, publication status, or date limit was used. Additionally, we performed a gray literature search through (1) manual hand search of bibliographies of identified randomized controlled trials and guidelines; (2) trials registries (Clinicaltrials.gov, World Health Organization WHO International Clinical Trials Registry Platform Search Portal, controlled-trials.com and the National Institutes of Health database of funded studies for ongoing or unpublished trials); and (3) conferences preceding and abstracts of the North American and European Endocrine Society and The Society of Adolescent Medicine and Health. Search alerts are set up for monthly notification, and the search will be repeated before the final manuscript submission to identify any new literature. We will contact the authors of unpublished work to establish eligibility and methodological quality of the study.

### Study selection

**Fig. 1:**
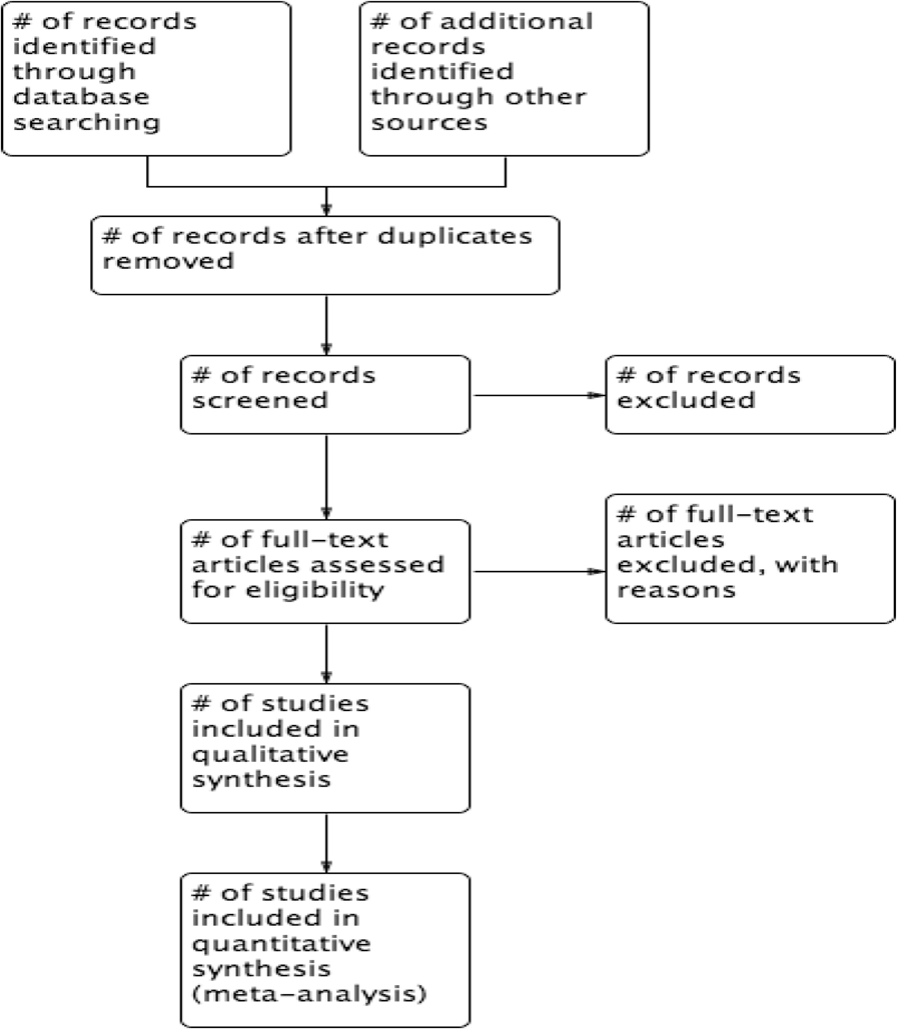
The primary selection process

Two reviewers (RA and IF) will independently and in duplicate screen the title and abstract available of identifiable articles to assess its eligibility. In case of disagreement, the full text will be retrieved and reviewed independently by one of the authors (EB), to resolve discrepancy. We will refer to inclusion and exclusion criteria during the screening process. Records of ineligible articles along with the reason for ineligibility will be saved for future reference. Eligible articles citations will be saved in EndnoteX6 library. We will include the PRISMA flow diagram demonstrating the search and screening process (Fig. 1). We will contact authors of primary studies during data extraction to provide any missing information.

### Data extraction

The study data will be collected in standardized online data extraction forms (Google forms) according to pre-specified instructions. The data extraction form will include information pertaining to study background, language of publication, country, funding sources, confirm study eligibility, participant ages, PCOS diagnostic criteria, the study design, number of intervention groups, intervention details, number of participants allocated to each intervention group, randomization, concealment of allocation, blinding, length of follow up, analysis type, outcome definition, unit of measurement, ascertainment of the outcome, estimate of intervention effect with confidence interval, and missing follow up data. When studies measure outcomes at more than one time point, we will collect results for the last measurement point in the study. The data extraction form will be pilot tested by all reviewers independently before its use. Four reviewers will perform data extraction (RA, IF, EB, BD), working in pairs independently and in duplicate. In case of disagreement in assessing the methodological quality of the study, we will try to resolve it by consensus. If consensus cannot be reached, a third designated reviewer from the team will be involved.

### Assessment of risk of bias in included studies

Two independent reviewers will assess each included study for risk of bias using the modified Cochrane handbook for systematic reviews of interventions tool [37], which assesses six elements: (1) sequence generation, (2) allocation concealment, (3) blinding of participants, personnel and outcome assessors, (4) completeness of follow up, (5) selective outcome reporting, and 6) presence of other biases. Each domain will be assigned a score of “low risk,” “high risk,” or “unclear risk.” We will further categorize the “unclear risk” to “probably low risk” or “probably high risk” in order to give a better understanding of the unclear risk of bias score. We will rate the overall risk of bias score for each study as “high risk” if the study meets more than two criteria for high risk of bias, “moderate risk of bias” if the study meets one to two criteria for high risk of bias, and “low risk of bias” if the study does not meet any high risk of bias criteria [38].

### Standard direct comparisons

We will perform a pairwise meta-analysis using R software. Effect estimates and their 95th confidence interval (CI) will be calculated using risk ratio (RR) for binary outcomes and mean difference for continuous outcomes if they are reported using the same metrics; otherwise, estimates reported using different metrics will be converted into standardized mean difference (SMD). We will pool all direct evidence using random-effect meta-analysis with the maximal likelihood (ML) estimator [39]. We will assess for heterogeneity by estimating the variance between studies using the chi-square test and quantify it using the *I*^2^ test statistic. We will interpret the *I*^2^ using the thresholds set forth by the Cochrane Collaboration [37].

### The network meta-analysis

Given that many of the treatment combinations available to treat PCOS were not compared in head-to-head studies, a network meta-analysis (NMA) will be necessary to provide effect estimates for all indirect comparisons [33]. We will perform a multiple treatment comparison to estimate the pooled direct, indirect, and the network estimates (mixed evidence from direct and indirect estimates) for all PCOS interventions on outcomes if the assumptions of homogeneity and similarity are judged to be reasonable. Effect estimates will be presented along with their corresponding 95 % credibility intervals (CrIs); these are the Bayesian analog of 95 % CIs. However, mixed evidence will only be used if the consistency assumption is met.

We will fit a Bayesian random-effect hierarchical model with non-informative priors using vague normal distribution (mean 0, variance 10,000) and adjusting for correlation between effects in multi-arm trials. We will generate posterior samples using Markov Chain Monte-Carlo (MCMC) simulation technique running the analysis in four parallel chains. We will use a series of 100,000 burn-in simulations to allow convergence and then a further 20,000 simulations (succeeding 50,000 simulations saved at an interval of 10 in each chain) to produce the outputs. We will assess model convergence using Gelman and Rubin diagnostic test [40]. The Bayesian model provides flexibility for moderate levels of treatment heterogeneity, sampling variability, and incoherence [41]. This model introduces a random effect representing any changes in the observed treatment effect that may be due to the comparison being made [42]. We will interpret variability in this random effect as incoherence [41]. We will use the node-splitting method to detect incoherence between direct and indirect evidence within a closed loop as well as identify loops with large inconsistency [42, 43]. We will measure the goodness-of-fit of the model using the deviance information criterion (DIC) [42].

To ensure interpretability of the NMA results, we will present the network geometry, the results with probabilistic statements, and also the estimates of interventions effects and corresponding 95 % CrIs, as well as forest plots. We will first rank the intervention and report each interventions’ probability of ranking first (being the best treatment) as well as the surface under the cumulative ranking curve (SUCRA) values [44]. High SUCRA values are expected for the best treatments, and low SUCRA values are expected for the worst treatments.

The above analysis assumes that the interventions are competing (suitable when most components forming an intervention are pharmaceutical and hence cannot go together), so that each combination is considered to be a separate treatment. For example, a combination of components of metformin, flutamide, and exercise forming an intervention and another combination of metformin and exercise forming another intervention are treated as competing interventions and assesses whether one combination is better than another. However, it is possible to perform the network meta-analysis treating these combinations as complex intervention [45]. Such an analysis fits a similar hierarchical regression model but considers multiple components of an intervention as dummy variables in the same model. Hence, the analysis allows the estimation of the effects and ranking of a combination of all possible and appropriate components. Thus, it is possible to explore such a combination that could have been the best for the treatment of PCOS but has never been tested before in any trial. Further, such an analysis allows the assessment of additive or multiplicative (interaction) effects between two or more components if sufficient data are available. We will re-analyze the data under complex intervention approach as well [45] to assess if there exists a potentially better combination of components which have been ever or never assessed.

We will perform the Bayesian network meta-analysis in JAGS (version 3.4.0) or WinBUGS software (version 1.4.3, MRC Biostatistics Unit, Cambridge, UK) interfacing through R software.

### Meta-regression

In case there is significant heterogeneity and inconsistency, we will use meta-regression to explain the heterogeneity, provided we have enough data to do so; otherwise, we will perform subgroup analyses. We will perform meta-regression using study level covariates: methodological quality (high risk of bias versus low risk of bias), participant’s average age, BMI status (obese and/or overweight BMI ≥25 kg/m^2^ versus normal <25 kg/m^2^), homeostatic model assessment (HOMA-IR) (high and moderate ≥3 versus low <3), medication dose, length of treatment (≥3 months versus <3 months), use of ultrasound to document polycystic ovaries (used versus not used), and studies that included young adults versus adolescents only to examine the improvement or change in model fit after covariates are included into the model. We will also perform a subgroup analysis to evaluate the effectiveness of different oral formulations of contraceptive pill on changes of hirsutism and acne scores.

### Rating the confidence in estimates of the effect in NMA

**Fig. 2:**
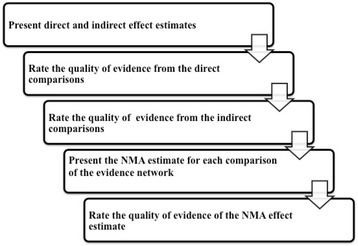
The quality assessment flow diagram

The confidence in the estimates (quality of evidence) for each reported outcome will be assessed independently by two reviewers (RA, IF) using the Grading of Recommendations Assessment, Development, and Evaluation Working Group (GRADE Working Group) approach; see Fig. 2 for the flow of quality assessment [46]. The quality of evidence is categorized by GRADE into four levels: high quality, moderate quality, low quality, and very low quality. For the direct comparisons, we will assess and rate each outcome based on the five GRADE categories: risk of bias, imprecision, inconsistency, indirectness, and publication bias [47].

For the assessment of confidence in the estimates obtained in the NMA, we will use the recent approach recommended by the GRADE working group [48]. We will assess and rate the confidence in all the indirect comparisons, if available, obtained from first order loops following the five GRADE categories used for assessing the direct comparisons in addition to the intransitivity assessment. Then, we will rate the confidence in each NMA effect estimate using the higher quality rating when both direct and indirect evidence are present. However, the estimate can be rated down for incoherence [48].

## Discussion

PCOS treatment in adolescents poses clinical challenges to patients and physicians. To our best knowledge, our study will be the first NMA in adolescents to investigate the effectiveness and safety of using metformin and OCP as monotherapy as well as in combination with pioglitazone, spironolactone, flutamide, or lifestyle interventions.

Our planned approach for this review has many strengths. We will implement a wide search strategy that included published and unpublished work. As adolescent women share some similar physiology with adult women and in an effort to overcome publication bias, we also plan to include studies that included adolescents and young adults. Additionally, we aim to report on many patient important outcomes as inferred from previous qualitative research. Similar to previous systematic reviews in adults with PCOS, we anticipate that we will identify studies which use different definitions of PCOS, various definitions for outcome measures of interest, and small sample sizes [49]. These factors may pose potential limitations to our study.

We hope that this review will provide hierarchical evidence to improve patient care, help unify the treatment approaches among specialists, and encourage research for new therapeutic options.

## Appendix

**Table 2:** Search strategy: Ovid MEDLINE(R) in-process and other non-indexed citations, Ovid MEDLINE(R) Daily and Ovid MEDLINE(R) 1946 to January 29, 2015

1	exp Child/
2	child*.mp.
3	exp Adolescent/
4	exp Pediatrics/
5	peadiatric*.mp.
6	juvenile.mp.
7	teen*.mp.
8	exp Young Adult/
9	young.tw.
10	young adult*.mp.
11	adolescen*.mp.
12	youth.mp.
13	pediatric*.mp.
14	Or/1 - 13
15	exp Polycystic Ovary Syndrome/
16	polycystic ovar*.mp.
17	pcos.tw.
18	Stein-Leventhal syndrome.mp.
19	exp Hyperandrogenism/
20	exp Hirsutism/
21	Hirsutism.tw.
22	Hyperandrogen$.tw.
23	(polycystic ovar* adj3 (syndrome or disorder or disease)).tw.
24	Or/15 - 23
25	exp metformin/
26	metformin.tw.
27	glucophage.tw.
28	Glumetza.tw.
29	Riomet.tw.
30	fortamet.tw.
31	exp Contraceptives, Oral/
32	oral contraceptiv*.tw.
33	ocp.tw.
34	Yasmin.tw.
35	Desogen.tw.
36	diane.tw.
37	Mircette.tw.
38	Cyclessa.tw.
39	Ortho Tri-Cyclen.tw.
40	Ortho-Cyclen.tw.
41	exp spironolactone/
42	spironolactone.tw.
43	aldactone.tw.
44	exp Flutamide/
45	Flutamide.tw.
46	pioglitazone.mp.
47	actos.tw.
48	exp Life Style/
49	exp Health Promotion/
50	Diet Therapy/
51	exp Exercise/
52	Or/25-51
53	randomized controlled trial.pt.
54	Controlled clinical trial.pt.
55	randomized.ab.
56	placebo.ab.
57	clinical trials as topic.sh.
58	randomly.ab.
59	trial.ti.
60	Or/53 - 59
61	14 and 24 and 52 and 60
62	clomiphene.ti.
63	pregnancy.ti.
64	62 or 63
65	61 not 64
Embase 1974 to 2015 January 27	
1	child*.mp.
2	peadiatric*.mp.
3	juvenile.mp.
4	teen*.mp.
5	exp Young Adult/
6	young.tw.
7	young adult*.mp.
8	adolescen*.mp.
9	youth.mp.
10	pediatric*.mp.
11	exp pediatrics/
12	adolescent/
13	child/
14	1 or 2 or 3 or 4 or 5 or 6 or 7 or 8 or 9 or 10 or 11 or 12 or 13
15	exp ovary polycystic disease/
16	polycystic ovar*.mp.
17	PCOS.mp.
18	Stein-Leventhal syndrome.mp.
19	exp hirsutism/
20	Hirsutism.tw.
21	exp hyperandrogenism/
22	Hyperandrogen$.tw.
23	exp metformin/
24	metformin.tw.
25	glucophage.tw.
26	Glumetza.tw.
27	Riomet.tw.
28	fortamet.tw.
29	exp oral contraceptive agent/
30	oral contraceptiv*.tw.
31	ocp.tw.
32	Yasmin.tw.
33	Desogen.tw.
34	diane.tw.
35	Mircette.tw.
36	Cyclessa.tw.
37	Ortho Tri-Cyclen.tw.
38	Ortho-Cyclen.tw.
39	exp spironolactone/
40	aldactone.tw.
41	exp flutamide/
42	exp pioglitazone/
43	pioglitazone.tw.
44	actos.tw.
45	exp lifestyle/
46	exp health promotion/
47	exp diet therapy/
48	exp exercise/
49	23 or 24 or 25 or 26 or 27 or 28 or 29 or 30 or 31 or 32 or 33 or 34 or 35 or 36 or 37 or 38 or 39 or 40 or 41 or 42 or 43 or 44 or 45 or 46 or 47 or 48
50	random.tw.
51	placebo.mp.
52	Double blind.tw.
53	Single blind.tw.
54	50 or 51 or 52 or 53
55	pregnancy.ti.
56	clomiphene.ti.
57	15 or 16 or 17 or 18 or 19 or 20 or 21 or 22
58	55 or 56
59	14 and 49 and 54 and 57
60	59 not 58
Cochrane central: search up to 30/01/2015 20	
#1 MeSH descriptor: [Adolescent] explode all trees	
#2 MeSH descriptor: [Child] explode all trees	
#3 MeSH descriptor: [Young Adult] explode all trees	
#4 young adult.tw	
#5 adolescen*.tw	
#6 #1 or #2 or #3 or #4 or #5	
#7 MeSH descriptor: [Polycystic Ovary Syndrome] explode all trees	
#8 Stein Leventhal Syndrome or Polycystic Ovar* or PCOS. tw	
#9 MeSH descriptor: [Hirsutism] explode all trees	
#10 MeSH descriptor: [Hyperandrogenism] explode all trees	
#11 hyperandrogen*. tw	
#12 Hirsutism. tw	
#13 #6 or #7 or #8 or #9 or #10 or #11 or #12	
#14 metformin or glucophage or pioglitazone or oral contraceptive pill or OCP or Ortho Tri-Cyclen or Ortho-CyclenCyclessa or Mircette or Yasmin or diane or Desogen or Flutamide or spironolactone or aldactone. tw	
#15 MeSH descriptor: [Metformin] explode all trees	
#16 MeSH descriptor: [Contraceptives, Oral, Hormonal] explode all trees	
#17 MeSH descriptor: [Spironolactone] explode all trees	
#18 MeSH descriptor: [Flutamide] explode all trees	
#19 MeSH descriptor: [Life Style] explode all trees	
#20 #14 or #15 or #16 or #17 or #18 or #19	
#21 #7 and #13 and #20 in Trials	

## Abbreviations

BMI: body mass index
DIC: deviance information criterion
GRADE: Grading of Recommendations Assessment, Development and Evaluation
NIH: National Institutes of Health
NMA: network meta-analysis
OCP: oral contraceptive pill
PCOS: polycystic ovarian syndrome
RCT: randomized controlled trials
T2DM: type two diabetes mellitus
